# Category learning difficulties in ADHD across modalities and multiple learning systems

**DOI:** 10.3758/s13423-025-02743-0

**Published:** 2025-09-25

**Authors:** Casey L. Roark, Yael Ben-Anat, Yafit Gabay

**Affiliations:** 1 Department of Psychology, University of New Hampshire, Durham, NH USA; 2 Department of Special Education and the Edmond J. Safra Brain Research Center for the Study of Learning Disabilities, University of Haifa, Mount Carmel, 31905 Haifa, Israel

**Keywords:** ADHD, Category learning, Procedural learning, Reinforcement learning, Modality

## Abstract

Attention-deficit/hyperactivity disorder (ADHD) has been associated with suboptimal functioning of both the prefrontal cortex and the striatum. These abnormalities may impede the acquisition of perceptual categories, important for fundamental abilities such as object recognition and speech perception. While prior research has shown that children with ADHD perform comparably to neurotypical peers in visual category learning despite using suboptimal strategies, much remains unknown about how adults with ADHD acquire perceptual categories, where more mature functions may shape learning processes differently than in childhood. To address this gap, we investigated auditory and visual category learning in adults with ADHD compared with neurotypical controls. Specifically, we focused on two types of category structures: rule-based categories, which are believed to rely on hypothesis-testing mechanisms mediated by the prefrontal cortex, and information-integration categories, thought to depend on reinforcement learning processes governed by the striatum. Our findings revealed consistent impairments in both rule-based and information-integration category learning among adults with ADHD across sensory modalities. Furthermore, category learning performance was negatively associated with ADHD symptom severity. Computational modeling analyses showed that individuals with ADHD were slower to adopt optimal learning strategies than their neurotypical counterparts, regardless of the category type or sensory modality. These findings point to disruptions in multiple learning systems in young adults with ADHD that extend across sensory modalities and arise from impairments in domain-general mechanisms.

**Supplementary Information:**

The online version contains supplementary material available at 10.3758/s13423-025-02743-0.

Attention-deficit/hyperactivity-disorder (ADHD) is a neurodevelopmental disorder that is characterized by a persistent and pervasive pattern of inattention and/or hyperactivity-impulsivity that interferes with functioning or development (American Psychiatric Association, 2013). Although ADHD is typically diagnosed in childhood, a substantial proportion of individuals continue to exhibit symptoms into adulthood (Di Lorenzo et al., 2021; Newcorn et al., 2024). Understanding the cognitive deficits associated with ADHD across developmental stages is critical, as these impairments can present lifelong challenges. In this study, we examine possible cognitive challenges in young adults with ADHD by examining perceptual category learning.

## Perceptual category learning

Perceptual category learning is a core cognitive function that underpins critical abilities such as speech perception (Holt & Lotto, 2010) and object recognition (Palmeri & Gauthier, 2004). A prominent framework for understanding this process, the Competition between Verbal and Implicit Systems (COVIS), posits the involvement of multiple distinct cognitive and neural systems in category learning: an explicit, hypothesis-testing system and an implicit, reinforcement-based system (Ashby et al., 1998; Waldron & Ashby, 2001). The explicit system primarily involves prefrontal cortex and heavily relies on working memory and executive attention and is optimal for learning rule-based (RB) categories where distinctions among categories can be easily described by verbalizable rules. The implicit system primarily mediated by the striatum, involves dopamine-reliant procedural learning of stimulus–response associations (Ashby & Todd Maddox, 2011), and is optimal for learning information-integration (II) categories where distinctions among categories are not easily described by verbalizable rules.

## Perceptual category learning in ADHD

There are reasons to expect that RB and II category learning may be disrupted in ADHD. ADHD has been linked to impairments in executive functions (Alderson et al., 2013; Boonstra et al., 2005; Chan et al., 2009; Diamond, 2013; Papadopoulos et al., 2005; Romine et al., 2004), which are thought to arise due to dysfunction in the prefrontal cortex (Barkley, 1997). Impairments in selective attention and executive functioning may interfere with the ability to learn RB categories mediated by the explicit learning system. In neurotypical populations, RB learning is strongly linked to working memory and selective attention (Zeithamova & Maddox, 2006). Evidence further supports this connection: tasks requiring interference control of attention, such as the Stroop and Simon tasks, have been tied to RB category learning performance (Waldron & Ashby, 2001) and are known to reveal impairments in ADHD populations (van Mourik et al., 2009). Additionally, adults with ADHD perform poorly on the Wisconsin Card Sorting Task, a paradigm dependent on rule-based processes akin to those required for RB learning (Arán Filippetti et al., 2020). Together, these findings suggest that individuals with ADHD may be impaired at RB category learning, driven by inefficiencies in the explicit learning system.

ADHD is associated not only with deficits in executive functions but also with impairments in implicit and procedural learning across motor and visual domains (Adi-Japha et al., 2011; Ballan et al., 2022; Barnes et al., 2010; Domuta & Pentek, 2003; Duda et al., 2019; Fox et al., 2014; Frank et al., 2007; Gabay & Goldfarb, 2017; Gabay et al., 2018; Takács et al., 2017). Moreover, ADHD is linked to altered reward processing (de Zeeuw et al., 2012; Luman et al., 2010) and impairments in reinforcement learning (Frank et al., 2007; Gabay & Goldfarb, 2017; Gabay et al., 2025; Huang-Pollock et al., 2014; Katabi & Shahar, 2024; Luman et al., 2009; Nissan et al., 2023; Sagvolden et al., 2005; Tripp & Alsop, 1999; Tripp & Wickens, 2008; Ziegler et al., 2016; Gabay et al. 2025), both of which are integral to learning via the procedural learning system (Ashby et al., 1998). At the neural level, individuals with ADHD exhibit functional and structural abnormalities in the striatum (Cubillo et al., 2012; Goodman et al., 2014; Sobel et al., 2010), a critical region for procedural learning. The abnormalities include reduced basal ganglia volumes (Castellanos et al., 2002; Sobel et al., 2010), altered striatal activation (Cubillo et al., 2012; Hart et al., 2013), and abnormal densities of dopamine transports (Dougherty et al., 1999; Krause et al., 2000). Collectively, these findings suggest that individuals with ADHD may be impaired at II category learning, driven by inefficiencies in the procedural learning system.

The relationship between ADHD and RB and II *visual* category learning in children with ADHD was investigated previously. Huang-Pollock et al. (2014) reported that children with ADHD performed comparably with their typically developing peers in both RB and II category learning tasks. However, closer examination revealed that children with ADHD employed markedly different *learning strategies*. Specifically, they tended to focus on a salient but irrelevant stimulus dimension when trying to learn RB categories, and they used random guessing strategies when learning II categories. This suggests that even if overall learning performance may appear similar between children with ADHD and their peers, the underlying cognitive strategies may differ.

## The present study

There are several gaps in the existing literature that are addressed in the current study. First, the prior study of category learning in ADHD focused on learning in children, when both RB and II category learning abilities are still developing (Reetzke et al., 2016; Roark & Holt, 2019; Zettersten et al., 2022). Further, the neural substrates supporting these learning processes undergo significant developmental changes. The prefrontal cortex undergoes substantial development between childhood and adulthood (Gogtay et al., 2004) and caudate volumes which are often reduced in children with ADHD, may normalize with age (Goodman et al., 2014), though some reductions may still be apparent (Montes et al., 2010; Proal et al., 2011; Seidman et al., 2011). Given such evidence, it remains unclear how category learning mechanisms operate in adults with ADHD.

Second, prior study of category learning in ADHD was limited to the visual modality, leaving open important questions about whether and how category learning in other sensory modalities may be affected. Emerging research among neurotypicals reveals that category learning involves both domain-general mechanisms and modality-specific processes (Roark et al., 2021, 2023). Therefore, examining category learning across sensory modalities is essential for understanding whether the learning disruptions in ADHD may arise due to impaired domain-general mechanisms (cognitive locus) or modality-specific deficiencies (perceptual locus).

In this study, we investigated RB and II category learning in young adults with ADHD and age-matched neurotypical controls, assessing performance across both visual and auditory modalities. To gain deeper insight into the mechanisms underlying learning performance, we examined not only overall learning outcomes but also the learning strategies employed by participants, using decision-bound computational modeling.

## Methods

### Participants

The sample included 61 participants within the age range of 20–35 years, 30 (11 men, 19 women) of whom were diagnosed with ADHD (*M*_age_ = 26.03 years, *SD* = 3.39) and 31 (11 men, 20 women) neurotypicals who served as a control group (*M*_age_ = 24.19 years, *SD* = 2.23). They were all university students in the north of Israel, mainly from families of middle to high socioeconomic backgrounds and were native Hebrew speakers of Israeli Jewish ethnicity. The inclusion criteria for the ADHD group included (1) a formal diagnosis of ADHD by an authorized clinician; (2) positive screening for ADHD based on the adult ADHD self-report scale (ASRS; namely, a score ≥51,Konfortes, 2010); (3) the lack of a formal diagnosis of a comorbid developmental disorder such as developmental dyslexia; (4) a cognitive ability score within the normal range >10th percentile Raven score (Raven & Court, 1998). Based on this criteria, one ADHD participant was excluded from the analysis. Therefore, the final sample included 29 participants with ADHD (11 men, 19 women, *M*_age_ = 26.2 years). The control group was composed of individuals with no history of learning disabilities who exhibited no difficulties in attentional skills (e.g., did not receive a positive score of ADHD based on the ASRS) and was matched in gender, and nonverbal intelligence (assessed by the Raven test; Raven & Court, 1998) to the ADHD group. Written informed consent was obtained from all participants, and the study was approved by the institutional review board of the University of Haifa and was conducted following the Declaration of Helsinki. All participants completed a battery of cognitive assessments evaluating general intelligence (Raven & Court, 1998), as well as reading (Shatil, 1997) and short-term memory (as assessed by the digit span test; Wechsler et al., 2008). Results showed no significant differences between the ADHD and control groups in intelligence (Raven’s score: *p* = .17), reading (words per minute: *p* = .13), or verbal working memory (digit span: *p* = .13). As the ADHD self-report score was used as inclusion criteria for both groups, the ADHD group scored significantly higher (more impairment) on the ASRS questionnaire (*p* < .001; Supplementary Table 1). The control group was also slightly significantly younger than the ADHD group, *t*(58) = −2.42,* p* = .02) but learning differences across groups did not change when controlling for age (Supplementary Table 2).

## Stimuli

**Fig. 1:**
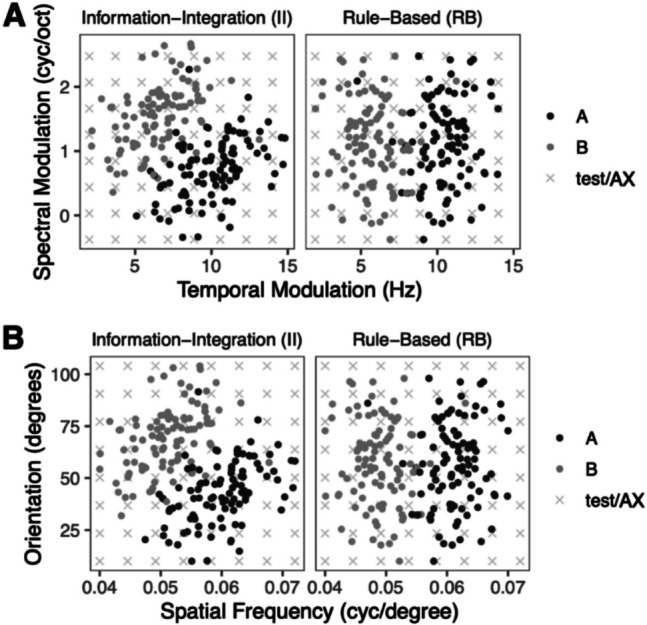
Category distributions. **A** Auditory category distributions. **B** Visual category distributions. Different shades denote stimuli from different categories. Xs denote stimuli used for generalization test and AX test

Participants learned two types of categories (II and RB) that have been extensively studied in prior research (Ashby & Maddox, 2005; Gabay et al., 2023; Roark et al., 2021). Each category type was comprised of two individual categories of stimuli sampled from a bivariate normal distribution (Fig. 1). The stimulus distributions were first generated in a normalized space and then were transformed into auditory and visual spaces as described below. There were 100 stimuli in each individual category and 200 for each category type (RB, II). The auditory stimuli were complex nonspeech ripples varying in spectral and temporal modulation with a duration of 1 s. Stimuli were WAV files generated using a custom MATLAB script and were amplitude matched at 70 dB. Stimuli varied across a range of spectral modulation (−0.38–2.67 cycles/octave) and temporal modulation (2.0–14.8 Hz). The visual stimuli were Gabor patches (sine wave gratings in which contrast is modulated by a circular Gaussian filter) varying in spatial frequency and orientation. Stimuli varied across a range of spatial frequency (0.04–0.072 cycles/degrees) and orientation (10–104 degrees). Distributional information for individual categories is shown in Table 1. The specific auditory and visual dimensions were chosen to be reasonably similar as they are all fundamental to perception in the key modality (Visscher et al., 2007; Woolley et al., 2005). A recent study suggests that these types of visual and auditory categories result in similar learning performance in neurotypical adults (Roark et al., 2021). Further, to simulate real-world category learning challenges, all category types were built up such that some level of overlap existed between categories. Therefore, perfect performance would not be possible, yet a maximum performance of 95% could be achieved for both RB and II categories (10/200 stimuli fall on the opposite side of the category boundary). Prior research shows that such overlap allows for effective learning of both category types (Ell & Ashby, 2006; Roark & Holt, 2018).

**Table 1:** Category Distribution Information

Auditory			
Category	Temporal Modulation	Spectral Modulation	Covariance
Information-Integration (A)	10.0 (2.13)	0.72 (0.49)	0.43
Information-Integration (B)	6.62 (2.02)	1.53 (0.51)	0.43
Rule-Based (A)	10.42 (1.58)	1.14 (0.66)	0.062
Rule-Based (B)	5.59 (1.58)	1.14 (0.66)	−0.062
Visual			
Category	Spatial Frequency	Orientation	Covariance
Information-Integration (A)	0.060 (0.0054)	43.2 (15.0)	0.034
Information-Integration (B)	0.051 (0.0051)	68.5 (15.8)	0.034
Rule-Based (A)	0.061 (0.0039)	56.7 (20.4)	0.0048
Rule-Based (B)	0.049 (0.0039)	56.7 (20.4)	−0.0048

## Procedure

### AX discrimination

To account for any potential differences across groups in sensory processing, participants discriminated pairs of sounds/visual stimuli drawn from the same stimulus space combining both dimensions (orientation and spatial frequency) as the categories prior to category learning. The specific exemplars were not experienced later in training and are approximately equidistant in perceptual space (Fig. 1, Xs). Participants responded with a key press, with no feedback, to every pairwise combination (275 ms ISI; 10 randomized repetitions; 1:1 same/different AX trials).

### Category learning

Participants completed all four tasks, learning both types of categories (RB, II) in both modalities (auditory, visual). To minimize potential carryover effects, participants completed the tasks in two sessions separated by 1 week. In each session they completed one visual and one auditory task. The order of modality was counterbalanced across participants yet remained constant within the two sessions. The order of tasks (RB, II) was counterbalanced across participants. Differences in task order did not influence learning differently across groups (see Supplemental Materials).

The tasks were identical and involved categorizing stimuli into one of two categories by pressing specific keys on a keyboard (1, 2). The association between the categories and the key responses was counterbalanced across participants. Each task had eight blocks, with each block containing fifty trials, for a total of 450 trials per task. After each training task, participants completed a generalization test in which they had to categorize new exemplars while receiving no feedback.

On each trial, the auditory or visual stimulus was presented for 1 s, followed by a presentation of a question mark presented until participants responded. Exemplars were sampled randomly but evenly across both categories for each block from the total pool of stimuli in a way that after four learning blocks the whole category space was presented. All participants were exposed to the same category distributions as depicted in Fig. 1. Feedback on whether the response was correct or incorrect was given 500 ms after each response and was presented for 1 s. Participants were instructed to answer as accurately and fast as possible but there was no time limit to their response. There was a 1-s ITI. In the generalization test, participants experienced novel stimuli from an 8x8 grid they had not encountered during training (same as AX stimulus grid; Fig. 1, Xs) and did not receive feedback for their response, but otherwise the trial procedure was the same.

## Computational analysis of learning strategies

Participants' learning strategies were assessed with decision-bound computational models (Ashby, 1992; Ashby & Gott, 1988; Ashby & Maddox, 1993). Similar to previous studies (e.g., Gabay et al., 2023; Maddox & Ashby, 2004) three classes of decision-bound models were applied to analyze category response data: hypothesis-testing models, a procedural-based model, and guessing models.

### Hypothesis-testing models

Hypothesis-testing models assume that participants use linear decision boundaries orthogonal to the stimulus dimensions to separate the stimuli into categories. The hypothesis-testing models assumed that participants use a unidimensional rule along a single dimension to separate the stimuli into categories (e.g., Category A has low temporal modulation, Category B has high temporal modulation). We fit separate models that assumed participants used either stimulus dimension (e.g., temporal or spectral modulation in the auditory modality, spatial frequency or orientation in the visual modality) for unidimensional models. We also fit different versions of the models that differ in their assignment of category response to a specific region of space (e.g., Category A has low temporal modulation and B has high or Category A has high temporal modulation and B has low).

The unidimensional rule hypothesis-testing models have two free parameters—one for the placement of the decision boundary along the relevant dimension and one for perceptual and criterial noise. The unidimensional rule hypothesis-testing model is optimal for the RB categories (auditory: temporal modulation; visual: spatial frequency).

### Procedural-based model

The procedural-based model we used was the General Linear Classifier (GLC; Ashby et al., 2007; Ashby & Waldron, 1999). The GLC model employs a linear decision boundary that is nonorthogonal (i.e., diagonal) to the stimulus dimensions to separate the categories. The GLC model has three free parameters—one for the slope of the boundary, one for the intercept of the boundary, and one for perceptual and criterial noise. To ensure reasonable fit, we fit two versions of the procedural-based model that assume different assignments of responses to regions of space. The procedural-based model is optimal for the II categories.

### Guessing models

The final set of models assumes that participants guess the category identity on each trial. One of the guessing models assumed that participants responded with equal probability for different category labels, while two additional models accounted for potential biases in responses, where participants were more likely to choose one label over the other. The random models have one free parameter—the probability of responding Category “A,” where the probability of responding Category “B” is 1 minus the probability of responding Category “A.”

### Model fitting and selection

All models were applied to each block of each participant’s data. The models were fitted using maximum likelihood procedures (Wickens & Sandry, 1982) separately for each block. To determine the best-fit model for each participant and block, we employed the Bayesian information criterion (BIC) as a measure of goodness-of-fit. The BIC is calculated as BIC = *r*lnN – 2lnL, where *r* represents the number of free parameters, *N* denotes the number of trials in a specific block for a particular subject, and *L* represents the likelihood of the model given the data (Schwarz, 1978).

Decision bound computational modeling was performed in Python (Version 3.7.4). Analyses of computational modeling results were performed in R (Version 4.3.1), using the following packages: *rstatix* (Version 0.7.2; Kuznetsova et al., 2017), *lme4* (Version 1.1.34; Bates et al., 2015), *lmerTest* (Version 3.1.3; Kuznetsova et al., 2017). Figures were created in R using the *ggplot2* (Version 3.3.5; Wickham et al., 2016) and *ggthemes* (Version 5.1.0; Arnold, 2024) packages.

## Power analysis

We used G*Power software (Faul et al., 2007) to perform a post hoc sensitivity analysis, which reveals the smallest effect that could have been reliably detected with 80% power assuming α = 0.05 with our sample size (*N* = 60). The analysis revealed we could reliably detect a small effect size (*f* = 0.12) for the full mixed-design analysis of variance (ANOVA; 2 groups × 2 modalities × 2 tasks × 8 blocks) and a small to medium effect size (*f* = .25) for the mixed-design ANOVA used in the majority of our analyses (2 groups × 2 modalities × 2 tasks). We take a linear mixed-effects modeling approach to compare performance across conditions. Because these models afford higher power than their ANOVA counterparts, this provides a conservative estimate of minimal detectable effect size.

## Results

### Prelearning AX discrimination

There were no group differences in sound discrimination, but there were slight differences in visual discrimination (ADHD: *M* = 0.89, *SE* = 0.09; control: *M* = 0.84, SE = 0.09), *t*(58) = 2.74, *p* = .008. However, these group differences were not related to changes in performance (Supplementary Table 3).

## Category learning

**Fig. 2:**
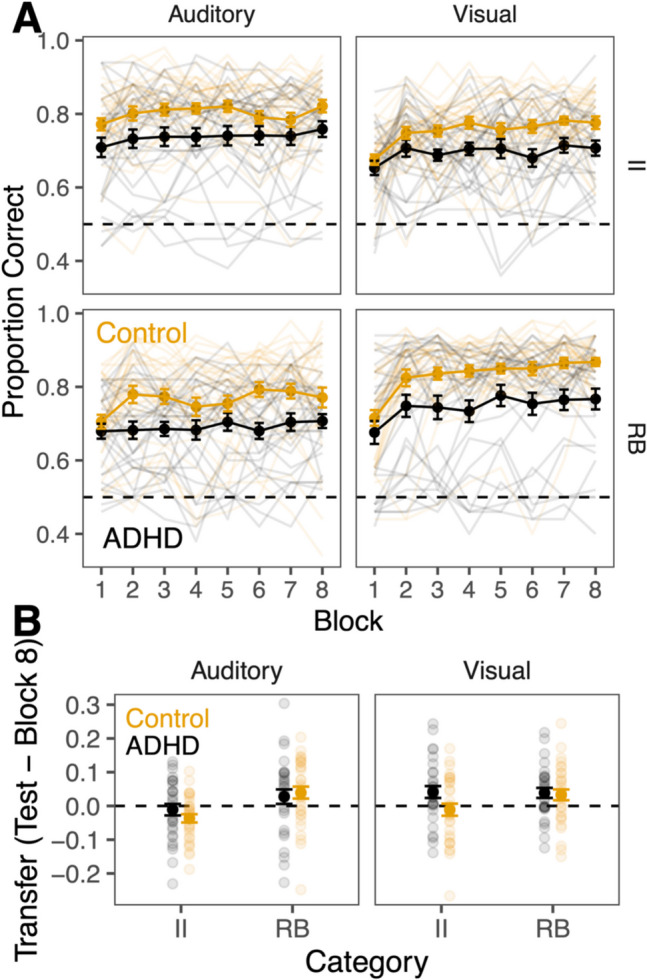
Category learning performance. Error bars represent standard error of the mean. Dashed line represents chance-level performance. Rule-Based (RB) and Information-Integration (II) categories are abbreviated. **A** Category learning performance across blocks. **B** Category learning performance in the generalization test. Lighter points and lines represent individual participant performance and darker points and lines represent the means across participants. (Color figure online

We compared accuracy across groups (ADHD, control), modalities (visual, auditory), category type (RB, II), and training blocks (1–8) using a linear mixed effects model with a random intercept for each participant. The ADHD group, auditory modality, and II categories were treated as reference groups and block was treated as a continuous variable. Overall, for the auditory II categories, the ADHD group performed significantly worse than the control group (Fig. 2A, β = 0.075, *p* = .0048). The ADHD group performed worse than controls across tasks as the pattern did not differ across RB categories or modalities (absolute value of all βs for all interactions with group < 0.042, *p* values > .11). While categorization accuracy generally improved across blocks, the overall linear effect of block was not significant (β = 0.0048, *p* = .079).

Performance in the visual modality was significantly worse than the auditory modality (β = −0.042, *p* = .030) and performance for RB categories was significantly worse than II categories (β = −0.042, *p* = .031). However, there was a significant interaction between modality and category (β = 0.0708, *p* = .0096). For RB categories, visual performance (*M* = 79%) was better than auditory (*M* = 73%), but for II categories, auditory performance (*M* = 77%) was better than visual (*M* = 73%). No other interactions were significant (full results are presented in Supplementary Table 4).

## Generalization transfer

Performance in the generalization test reflects the ability to categorize never-before-categorized stimuli without feedback. We compared transfer accuracy (test block minus final training block) across groups (ADHD, control), modalities (visual, auditory), and category type (RB, II) using a linear mixed effects model with a random intercept for each participant (Fig. 2B). Transfer performance in the visual modality was significantly better than the auditory modality (β = 0.052, *p* = .024). There was no significant difference between ADHD and control groups (β = −0.026, *p* = .29) and no other significant effects or interactions (for full results, see Supplementary Materials, Table 5).

## ADHD symptom severity and learning performance

Based on the inclusion criteria, the ADHD group was specifically impaired in the self-report measure of ADHD symptom severity (higher ASRS scores) relative to controls. To understand whether self-reported symptom severity was related to general category learning abilities, we calculated the correlation between ASRS score and averaged category learning performance (across blocks, tasks, and modalities) across all participants. Overall, ASRS scores were *negatively* correlated with learning performance, *r*(58) = −0.46, 95% CI [−0.64, −0.24], *p* = .00019—more severe ADHD symptoms (higher ASRS scores) were associated with worse overall learning (Supplementary Fig. 1). Notably, a linear regression model with ASRS score as the only predictor (adjusted *R*^2^ = .20) accounted for more variance in category learning performance than a model with group as the only predictor (adjusted *R*^2^ = .18). These results may hint that poorer learning in the ADHD group may stem from similar sources as general ADHD symptoms.

## Computational analyses of learning strategies

**Fig. 3:**
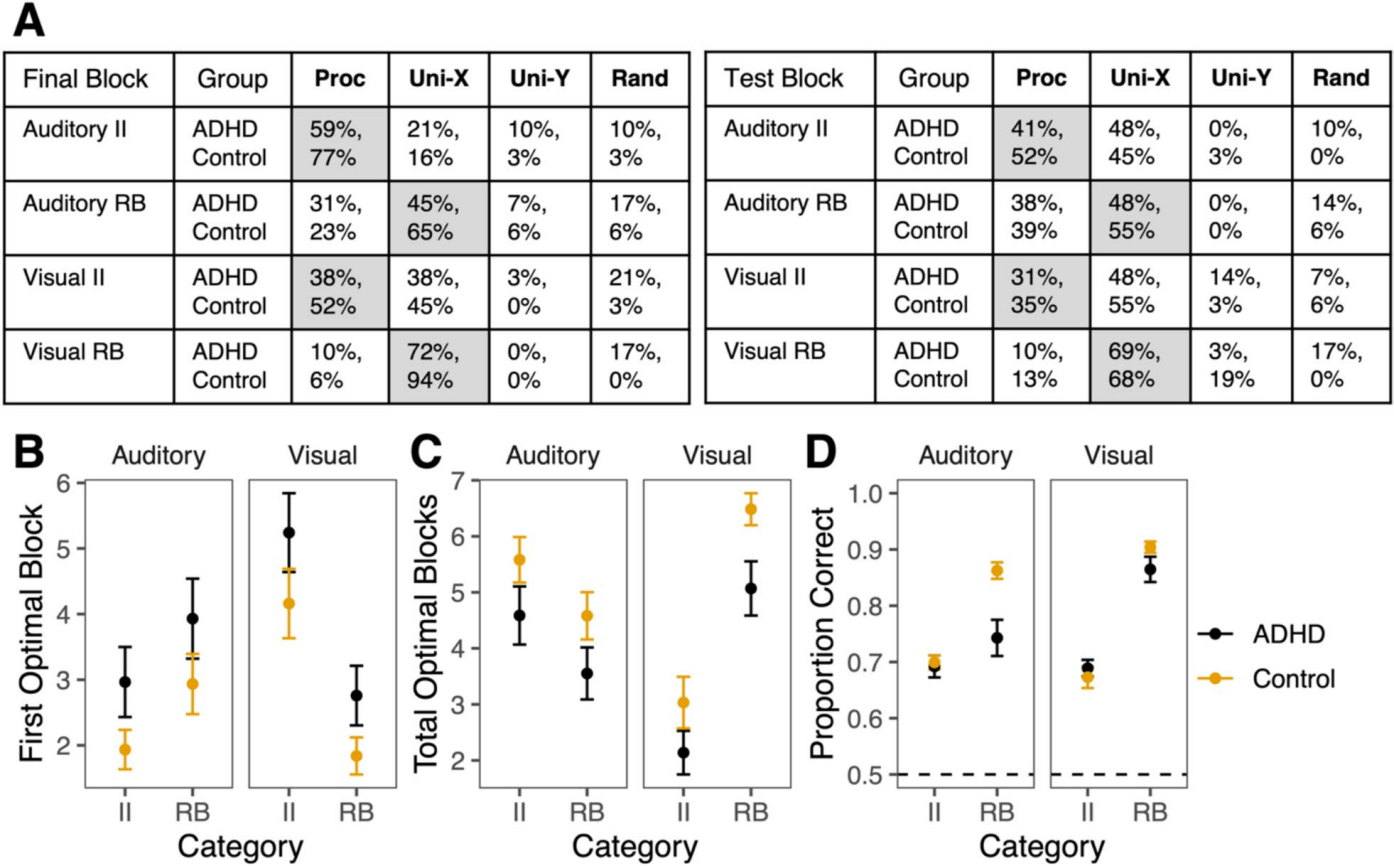
Learning strategies across groups. Error bars represent *SEM.* **A** Proportion of participants in ADHD (top row) and control (bottom row) groups using different strategies. Strategies are procedural (Proc), unidimensional (Uni) using the dimensions displayed on the *x* and *y* dimensions of Fig. 1 (X-auditory: temporal modulation; X-visual: spatial frequency; Y-auditory: spectral modulation; Y-visual: orientation), and random (Rand). Uni-X is the optimal strategy for the RB categories and procedural is the optimal strategy for the II categories, which is highlighted in gray.** B** First block in which learners used optimal strategy. **C** Total number of blocks in which learners used optimal strategy. **D** Among only participants using the optimal strategy, accuracy in the final training block. (Color figure online)

We compared learning strategies across groups in the final training block and the test block of each task (Fig. 3A). Overall, both groups used similar strategies across tasks (Fisher’s exact tests: all comparisons *p* values > .10 except visual RB task: final block: *p* = .047; test: *p* = .033). To better compare the ability to use task-optimal strategies over the course of learning, we compared three measures across groups, modalities, and categories using mixed-model ANOVAs (and *t* tests with fewer comparisons)—number of blocks it took participants to use the optimal strategy (Fig. 3B), the total number of blocks they used the optimal strategy (Fig. 3C), and when using the optimal strategy in the final training block, how efficient the strategy was for their performance (i.e., how accurate they were when they used the optimal strategy; Fig. 3D).

Regardless of category type or modality, controls (*M* = 2.72 blocks) used the optimal strategy significantly earlier than the ADHD group (*M* = 3.72 blocks), *F*(1, 58) = 5.21, *p* = .026,* η*_*G*_^*2*^ = 0.036. There was a significant modality-by-category interaction, *F*(1, 58) = 28.3, *p* < .0001,* η*_*G*_^*2*^ = 0.097, such that learners used the optimal strategy earlier in the auditory II (*M* = 2.43 blocks) and visual RB tasks (*M* = 2.28 blocks) and later in the auditory RB (*M* = 3.42 blocks) and visual II tasks (*M* = 4.68 blocks). Individuals with ADHD were less efficient at using the same optimal strategy in the auditory RB task compared with controls.

Regardless of category type or modality, controls (*M* = 4.92 blocks) used the optimal strategy in significantly more blocks than the ADHD group (*M* = 3.84 blocks), *F*(1, 58) = 8.52, *p* = .0050, *η*_*G*_^*2*^ = 0.051. There was also a significant modality-by-category interaction, *F*(1, 58) = 44.7, *p* < .0001, *η*_*G*_^*2*^ = 0.17, such that learners used the optimal strategy in more blocks in the auditory II (*M* = 5.10 blocks) and visual RB tasks (*M* = 5.80 blocks) and fewer blocks in the auditory RB (*M* = 4.08 blocks) and visual II tasks (*M* = 2.60 blocks).

In the final block of training, controls (*M* = 86%) using the optimal strategy in the auditory RB task had significantly higher accuracy than individuals with ADHD using the same strategy (*M* = 74%), *t*(17.1) = 3.39, *p* = .0035, *d* = 1.27. The groups did not differ in accuracy when using the task-optimal strategy in the auditory II, *t*(28.2) = 0.31, *p* = .76, *d* = 0.10, visual RB, *t*(28.1) = 1.60, *p* = .12, *d* = 0.48, or visual II tasks, *t*(24.9) = 0.66, *p* = .51, *d* = 0.25.

## Discussion

We examined RB and II category learning across auditory and visual modalities in adults with ADHD and neurotypical controls. Consistent with prior research in neurotypical populations (Roark, 2024; Roark & Chandrasekaran, 2023; Roark et al., 2021, 2023), participants in both groups demonstrated variability in their category learning performance. However, individuals with ADHD consistently underperformed compared with neurotypical controls across category types and modalities. Further, learning performance was negatively correlated with ADHD symptom severity across all participants, suggesting that learning impairments more broadly may stem from similar sources of general ADHD symptoms.

## Both explicit and procedural category learning are affected in ADHD

The pattern of results suggests that both explicit and procedural category learning present a challenge for young adults with ADHD. These findings resonate with prior research revealing impairments in young adults with ADHD when confronted with explicit (Arán Filippetti et al., 2020) or procedural category learning challenges (Gabay & Goldfarb, 2017; Gabay et al., 2018). Yet prior work also found that among *children* (8–12 years old) with ADHD, explicit/procedural visual category learning performance was not different from controls (Huang-Pollock et al., 2014). Our results demonstrate a different pattern in adults. Adults with ADHD performed significantly worse than neurotypical controls across all category types. These results suggest that the impact of ADHD on category learning may change over development, with impairments becoming more pronounced in adulthood. These adult-specific impairments are particularly relevant given that the prefrontal cortex is implicated in successful category learning (Schnyer et al., 2009) and is affected in adults with ADHD (Alexander & Farrelly, 2018). To better understand these developmental dynamics, future research should aim to replicate earlier findings in children with ADHD and examine category learning across extended developmental timescales, focusing on how the maturation of the PFC and executive functions interacts with learning processes in ADHD.

Considering learning mechanisms underlying category learning, Huang-Pollock et al. (2014) also found that children with ADHD more frequently used suboptimal learning strategies in both RB and II visual category learning compared with controls (Huang-Pollock et al., 2014). Our findings are broadly consistent with this pattern, highlighting notable differences in learning strategies between adults with ADHD and neurotypical controls across tasks. Regardless of category type or modality, individuals with ADHD were slower to adopt optimal learning strategies than their neurotypical counterparts. In the auditory RB task, adults with ADHD applied the optimal strategy less effectively, resulting in poorer overall performance as compared with controls. For the II tasks, individuals with ADHD demonstrated delayed adoption of the optimal strategy compared with controls.

Our results can also be interpreted through the lens of the COVIS model of category learning (Ashby et al., 1998). According to COVIS, we predicted that ADHD-related alterations in prefrontal (Arnsten, 2009) and striatal functioning (Goodman et al., 2014) would impair both frontally mediated RB and striatally mediated II category learning. Consistent with this prediction, significant learning impairments were seen across both RB and II categories in adults with ADHD. However, the source of these impairments remains uncertain.

One possibility is that RB and II learning deficits share a common origin in prefrontal cortex dysfunction and related executive function challenges in individuals with ADHD. For RB categories, the ADHD group may have difficulty disengaging from suboptimal rule strategies to find optimal rule strategies, requiring holding multiple hypotheses in mind and flexibly switching based on feedback. For II categories, the ADHD group may have difficulty switching control from the explicit rule-based system to the implicit procedural system. Evidence for prefrontal cortex involvement in this switching process comes from research showing that individuals with prefrontal lesions exhibit delays in adopting optimal strategies during II category learning (Schnyer et al., 2009). This interpretation aligns with a “single-system” perspective, which posits that executive functions supported by the prefrontal cortex play a critical role in category learning across RB and II categories, irrespective of whether the categories can be explicitly verbalized (Craig & Lewandowsky, 2012; Lewandowsky et al., 2012; Lloyd et al., 2019; Nosofsky & Kruschke, 2002). Alternatively, the observed impairments in RB and II category learning may arise from distinct underlying mechanisms, with RB deficits linked to prefrontal cortex dysfunction and executive functioning challenges (Barkley, 1997) and II deficits reflecting impairments in striatal function in ADHD (Ullman & Pullman, 2015). The COVIS model is primarily supported through neuropsychological and computational results and the recruitment of distinct neural systems during RB and II learning examined through fMRI has mixed results (Carpenter et al., 2016; Nomura et al., 2007). Because our results are behavioral, we cannot fully disentangle the dual and single systems interpretations of these results. Future work should disentangle these possibilities (with neuroimaging combined with computational modeling) to uncover the source or sources of RB and II category learning impairments in ADHD.

## The category learning deficiency of ADHD is domain-general

This work is the first to allow for direct comparison of auditory and visual category learning in ADHD. We found that, while performance was better in visual tasks compared with auditory tasks (at least for RB categories) and across both groups, learning was impaired in the ADHD group in both modalities and across category types, with relatively minor differences in auditory and visual category learning between the ADHD and neurotypical groups. The current results align with prior work that has demonstrated impaired visual procedural category learning impairments in adults with ADHD (Gabay & Goldfarb, 2017; Gabay et al., 2018) and extend this work to the auditory domain. The impaired RB category learning across visual and auditory modalities is consistent with prior work showing selective attention (Gomes et al., 2012; Laffere et al., 2021) and working memory (Kasper et al., 2012) impairments across sensory modalities among adults with ADHD. This could adversely influence their ability to filter sounds or visual stimuli or to preserve information over time and impact their RB category learning performance. Importantly, regardless of category learning challenge, our results demonstrate a modality-general explicit and procedural category learning deficits in ADHD, suggesting that the source of the impairments are likely cognitive rather than perceptual. Such findings particularly resonate with recent study suggesting that impaired high-level cognitive processes rather than low level perceptual processes are likely contribute to altered performance of adults with ADHD (Derawi et al., 2023).

We cannot completely exclude the possibility that visual perceptual differences contributed to impaired performance in the visual category learning tasks. The ADHD and control groups showed no significant difference in pre-training auditory discrimination, but the ADHD group had significantly worse pretraining visual discrimination compared with controls (see Supplemental Materials). This result leaves open the possibility that differences in visual category learning between groups may have been due to differences in visual sensory processing rather than cognitive processing. We are inclined to suggest that cognitive processes likely do still play a role in these visual learning differences across groups given that the discrimination performance differences were quite small, and that the ADHD group demonstrated similar learning impairments in the auditory modality where discrimination performance was not different across groups. However, future studies should aim to disentangle perceptual and cognitive processing problems in ADHD during learning. It is noteworthy that learning performance was more variable in the ADHD group (*SD* = 8.06%) relative to the control group (*SD* = 5.30%). As previously noted, category learning performance was negatively correlated with ADHD symptom severity. By definition, the ADHD group reported more severe ADHD symptoms than controls, but there was significant variability in both groups. Reported symptom severity was more variable in the ADHD group (*SD* = 11.43), compared with the control group (*SD* = 6.39). The increased variability in symptom severity may be related to the ADHD group’s increased variability in category learning.

## Conclusion

We found that adults with ADHD have modality-general and category-general learning impairments, manifested in worse learning and generalization performance and more suboptimal strategy use compared with neurotypical controls. These findings suggest that learning impairments in ADHD likely stem from cognitive, rather than perceptual, sources, affecting auditory and visual performance in similar ways. Our results can be interpreted through a dual systems framework, where prefrontal and striatal impairments in ADHD affect learning across multiple learning systems, though importantly, our results may also be interpreted through a single systems framework, where prefrontal impairments in ADHD affect learning outcomes broadly.

## Supplementary Information

Below is the link to the electronic supplementary material.

Supplementary file1 (DOCX 2.27 MB)

## Data Availability

The data is available via the Open Science Framework (https://osf.io/wzxac/). The experiments were not preregistered.
