# Supplementary material for: Category learning difficulties in ADHD across modalities and multiple learning systems
Source: Psychon Bull Rev. 2025 Sep 25;32(6):3300–11. doi: 10.3758/s13423-025-02743-0 (PMC12627133; doi:10.3758/s13423-025-02743-0)
Supplement: Supplementary file 1 — Supplementary file1 (DOCX 2.27 MB) [file 13423_2025_2743_MOESM1_ESM.docx]

**Supplemental materials, Roark, Ben-Anat, & Gabay “**Category learning difficulties in ADHD across modalities and multiple learning systems**”, PBR**

**Supplementary Table 1**

*Performance of the ADHD and Control Groups on the Psychometric and Self-Report Tests*

| **Measurement** | **Control**  **M (SD)** | **ADHD**  **M (SD)** | ***t* -value** | ***p*** |
| --- | --- | --- | --- | --- |
| ASRS Score | 34.54 (6.39) | 66.13 (8.81) | -15.963 | <0.001 |
| Raven’s Score | 54.16 (3.62) | 52.48 (5.39) | 1.400 | .166 |
| Words per minute | 108.1 (15.8) | 101.55 (16.88) | 1.525 | .132 |
| Digit span | 10.71 (2.65) | 9.66 (2.52) | 1.549 | .126 |

**Do age differences contribute to group differences in category learning?**

Overall, participants in the Control group (*M* = 24.1, *SD* = 2.23) were slightly younger than participants in the ADHD group (*M* = 26.0, *SD* = 3.39; *t*(58) = -2.42, *p* = .02). To test for the possibility that this slight age difference could contribute to group differences in category learning, we conducted a linear regression on average training accuracy (across blocks, categories, and modalities) with fixed effects of group (ADHD as reference) and age. Overall, after controlling for age, the Control group still performed significantly better than the ADHD group (β = 0.069, *p* = .0011; **Supplementary Table 2**). As a result, it is unlikely that the differences in category learning between the ADHD and Control groups were due to age differences.

**Supplementary Table 2**

| **Fixed effects** | **Estimate** | **SE** | **p-value** |
| --- | --- | --- | --- |
| Intercept | 0.74 | 0.088 | < .0001 |
| Control Group | 0.069 | 0.020 | .0011 |
| age | -0.0011 | 0.0033 | .75 |

**Order effects**

We compared accuracies across RB and II categories within each modality depending on the order in which participants completed the tasks (i.e., RB then II or II then RB). Specifically, we examined the potential interaction between order and group (ADHD, Control). We ran linear regression analyses separately in the two modalities to enable examination of effects of order due to the fact that order was counterbalanced separately across modalities. For the visual modality, there was no effect of order (β = 0.-32, *p* = .25) and order did not significantly interact with group (β = -0.050, *p* = .21). For the auditory modality, there was an overall main effect of order (β = -0.077, *p* = .016) such that participants who completed the II task and then the RB task (*M* = 78%) had significantly higher average accuracies than participants who completed the RB task and then the II task (*M* = 73%). However, importantly, the effect of order did not significantly interact with group (β = 0.052, *p* = .24). This indicates that any differences in counterbalancing order did not significantly influence learning in a way that affected the groups differently.

**Pre-learning AX discrimination**

The ADHD and Control groups did not significantly differ in their ability to discriminate sounds (ADHD: *M*=0.85, SE =0.07; Control: *M*=0.87, SE=0.04; *t*(58) = 1.27, *p*=.20) though the Control group had significantly, though slightly, better discrimination for visual stimuli than ADHD (ADHD: *M*=0.89, SE =0.09; Control: *M*=0.84, SE =0.09; *t*(58) = 2.74, *p*=.008) before category training. Therefore, it is possible that these differences have contributed to impaired visual category learning performance of the ADHD group. To investigate this possibility, we conducted a linear mixed effects regression model to examine the relationship between visual AX discrimination accuracy and the final block accuracy in the visual RB and II tasks. We also examined fixed effects and interactions of group (ADHD vs. Control) with subject as a random effect. The ADHD group and II categories were used as reference groups.

Overall, there was a significant effect of visual discrimination accuracy on final block accuracy (β = 0.47, SE = 0.22, *p* = .031), indicating a positive relationship between these measures for the ADHD group in the II task. Importantly, there were no other significant effects or interactions, indicating that the relationship between these measures did not significantly differ across groups or tasks (absolute value of all βs < 0.29, *p*s > .27, see **Supplementary Table 3** below for full results). These findings suggest that visual perceptual differences may weakly relate to visual category learning but do not distinguish among groups or tasks. As a result, it is unlikely that the differences in performance in the visual II task between ADHD and Control groups were due to differences in baseline differences in visual discrimination ability.

**Supplementary Table 3**

*Visual Discrimination and Visual Category Learning*

| **Effect** | **Estimate** | **SE** | **p-value** |
| --- | --- | --- | --- |
| Intercept | 0.31 | 0.18 | .09 |
| Visual discrimination | 0.47 | 0.22 | .031 |
| Control Group | 0.12 | 0.36 | .73 |
| RB Category | 0.30 | 0.22 | .18 |
| Visual discrimination * Control Group | -0.087 | 0.40 | .83 |
| Visual discrimination * RB Category | -0.29 | 0.26 | .28 |
| Control Group * RB Category | 0.098 | 0.43 | .82 |
| Visual discrimination * Control Group * RB Category | -0.056 | 0.49 | .91 |

*Note.* Full results of the linear mixed effects regression with final block of the visual category learning tasks as the outcome variable, visual discrimination accuracy from AX task, group (ADHD vs. Control), and Category (RB vs. II) as fixed effects, and participant as a random effect. The ADHD group and II category were treated as reference groups.

**Supplementary Table 4**

*Category Learning Full Results*

| **Fixed Effect** | **Estimate** | **SE** | ***p*-value** |
| --- | --- | --- | --- |
| Intercept | 0.72 | 0.019 | < 2e-16 |
| Visual Modality | -0.042 | 0.019 | .03 |
| Block | 0.0048 | 0.0027 | .079 |
| Control Group | 0.075 | 0.026 | .0048 |
| RB Category | -0.042 | 0.019 | .031 |
| Visual Modality x Block | -0.000082 | 0.0038 | .98 |
| Visual Modality x Control Group | -0.043 | 0.027 | .11 |
| Block x Control Group | -0.0023 | 0.0038 | .54 |
| Visual Modality x RB Category | 0.071 | 0.027 | .0096 |
| Block x RB Category | -0.0011 | 0.0038 | .78 |
| Control Group x RB Category | -0.016 | 0.027 | .55 |
| Visual Modality x Block x Control Group | 0.0082 | 0.0053 | .12 |
| Visual Modality x Block x RB Category | 0.0058 | 0.0054 | .28 |
| Visual Modality x Control Group x RB Category | 0.041 | 0.038 | .28 |
| Block x Control Group x RB Category | 0.0055 | 0.0053 | .30 |
| Visual Modality x Block x Control Group x RB Category | -0.0051 | 0.0075 | .49 |

*Note.* Full results of the linear mixed effects regression with categorization accuracy as the outcome variable, Group (ADHD vs. Control), Block (1-8) and Category (RB vs. II) as fixed effects, and participant as a random effect. The ADHD group, auditory modality, and II categories were treated as reference groups and block was treated as a continuous variable.

**Supplementary Table 5**

*Generalization Transfer Full Results*

| **Fixed effects** | **Estimate** | **SE** | **p-value** |
| --- | --- | --- | --- |
| Intercept | -0.011 | 0.017 | 0.52 |
| Visual Modality | 0.052 | 0.023 | 0.024 |
| Control Group | -0.026 | 0.024 | 0.29 |
| RB Category | 0.039 | 0.023 | 0.094 |
| Visual Modality x Control Group | -0.027 | 0.032 | 0.40 |
| Visual Modality x RB Category | -0.041 | 0.032 | 0.21 |
| Control Group x RB Category | 0.038 | 0.032 | 0.24 |
| Visual Modality x Control Group x RB Category | 0.0091 | 0.045 | 0.84 |

*Note.* Full results of the linear mixed effects regression with transfer accuracy as the outcome variable, Group (ADHD vs. Control), Modality (visual, auditory) and Category Type (RB, II) as fixed effects, and participant as a random effect. The ADHD group, auditory modality, and II categories were treated as reference groups.

**Supplementary Figure 1**

*ADHD Symptom Severity and Overall Learning*


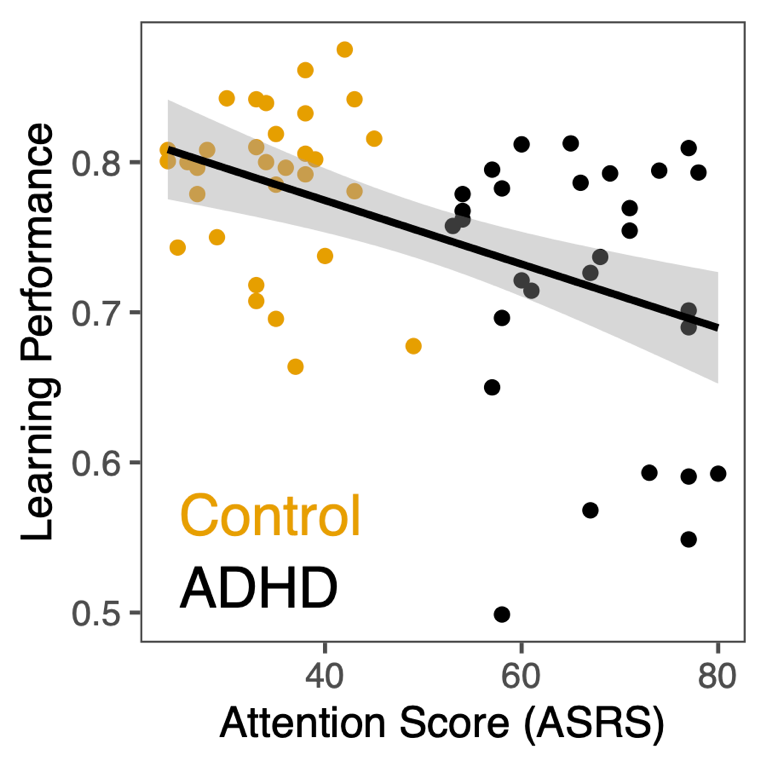


*Note.* Mean categorization performance is averaged across all blocks, categories, and modalities.
